# Hydrogen Gas: A Novel Type of Antioxidant in Modulating Sexual Organs Homeostasis

**DOI:** 10.1155/2021/8844346

**Published:** 2021-01-16

**Authors:** Yaxing Zhang, Haimei Liu, Jinwen Xu, Shuhui Zheng, Lequan Zhou

**Affiliations:** ^1^Department of Physiology, School of Basic Medical Sciences, Guangzhou University of Chinese Medicine, Guangzhou, Guangdong, China; ^2^Research Center for Translational Medicine, The First Affiliated Hospital, Sun Yat-sen University, Guangzhou, Guangdong, China

## Abstract

Sex is a science of cutting edge but bathed in mystery. Coitus or sexual intercourse, which is at the core of sexual activities, requires healthy and functioning vessels to supply the pelvic region, thus contributing to clitoris erection and vaginal lubrication in female and penile erection in male. It is well known that nitric oxide (NO) is the main gas mediator of penile and clitoris erection. In addition, the lightest and diffusible gas molecule hydrogen (H_2_) has been shown to improve erectile dysfunction (ED), testis injuries, sperm motility in male, preserve ovarian function, protect against uterine inflammation, preeclampsia, and breast cancer in female. Mechanistically, H_2_ has strong abilities to attenuate excessive oxidative stress by selectively reducing cytotoxic oxygen radicals, modulate immunity and inflammation, and inhibit injuries-induced cell death. Therefore, H_2_ is a novel bioactive gas molecule involved in modulating sexual organs homeostasis.

## 1. Introduction

Sex is a fundamental pleasure and quality-of-life issue [1, 2]. Sexual medicine represents one of the oldest medical specialties, and ancient civilizations had no qualms in discussing their sexual health and sexuality—an openness that has not pervaded through to modern life [3]. Although crucial to the survival of our species, human sex is clearly more complex than mere reproduction; sexual medicine is involved in endocrinology, gynecology, andrology, genetics, neurology, angiology, psychology, sociology, anthropology, and other related disciplines; human sex is cutting edge but bathed in mystery [1, 3, 4]. Traditionally, sexual intercourse or coitus, which is viewed as that female vagina receives the male erect penis, is at the core of sexual activities.

Coitus requires healthy and functioning vessels to supply the pelvic region in males and females, thus generating penile and clitoris erection and vaginal lubrication, respectively [5, 6]. Erection is a neurovascular event modulated by psychological and hormonal factors [5, 6]. Nitric oxide (NO), which is recognized as the main mediator of penile and clitoris erection, is synthesized and released from adjacent nonadrenergic noncholinergic (NANC) nerve endings *via* neuronal nitric oxide synthase (nNOS) and/or endothelial cells (ECs) *via* endothelial nitric oxide synthase (eNOS) upon mental and sensory stimuli *via* spinal reflex [5, 7, 8]. Upon its release, NO diffuses locally into adjacent cavernosal and vascular smooth muscle cells and binds with its physiologic receptor, soluble guanylyl cyclase (sGC) [5, 9]. This binding results in an enzyme conformational change, resulting in the conversion of guanosine triphosphate (GTP) to 3′,5′-cyclic guanosine monophosphate (cGMP) [5, 7, 10]. cGMP activates cGMP-dependent protein kinase (PKG); PKG phosphorylates several molecular targets, such as inositol 1,4,5-triphosphate (IP3) receptor, IP3 receptor-associated PKG substrate (IRAG), phospholamban (PLB), and calcium-activated potassium (BK_Ca_) channel [11, 12]; these result in decreasing free cytosolic calcium (Ca^2+^) concentration through increased its uptake into intracellular stores, such as the sarcoplasmic reticulum and mitochondria, and through increased efflux/decreased influx of Ca^2+^ across the plasma membrane, finally, induce relaxation of the vascular and cavernosal smooth muscle cells [5, 7, 13].

It is well established that the principal determinant of smooth muscle cell contraction is the concentration of intracellular free Ca^2+^, and myosin light chain (MLC) phosphorylation mediated by activated myosin light chain kinase (MLCK) in response to increased Ca^2+^ is the main pathway by which vasoconstrictor stimuli induce crossbridge cycling of myosin and actin filaments [14]. The secondary pathway for smooth muscle cell contraction that is not directly dependent on Ca^2+^ concentration, but rather mediating Ca^2+^ sensitization, is the RhoA/Rho kinase pathway [14]. In response to contractile stimuli, the small GTPase RhoA activates the downstream effector Rho kinase, which phosphorylates C kinase potentiated protein phosphatase inhibitor (CPI-17) and subsequently induces the interaction of CPI-17 with the catalytic subunit of myosin light chain phosphatase (MLCP), or phosphorylates the myosin-binding subunit (MBS, also known as MYPT1) of MLCP, thus inhibiting MLCP activity and promoting the phosphorylated state of the MLC and contraction [14–16]. Besides the removal of Ca^2+^ from the cytosol discussed above, PKG also induces the phosphorylation of GTPase RhoA to inhibit its activity, thus contributing to smooth muscle relaxation [11, 12]; additionally, PKG can directly interact with the myosin-binding subunit of MLCP to activate MLCP, thus inducing cGMP-dependent dephosphorylation of MLC and initiating the NO-mediated vasodilatation [11, 12, 17–19].

The resulting dilation of the cavernosal arterioles and sinuses results in increasing blood flow (driven by the force of the arterial blood pressure) and a subsequent rise in intracavernosal pressure, which subsequently activates a veno-occlusive mechanism to limit the outflow of blood and further increases the pressure inside the cavernosum [8, 20]. The erectile response ensues as the force of the elevated pressure expands the outer tunica albuginea of the penis, resulting in the increased penile length and diameter characteristic of erection [8]. Thus, at the onset of sexual stimulation, neuronal NO induced by neuronal depolarization and endothelial NO largely generated in response to shear forces brought on by increased blood flow in the penis serve, respectively, as a neurotransmitter initiating the erectile process and as a paracrine factor sustaining the full physiologic response [5, 8]. The molecular mechanisms for female clitoral engorgement and vaginal lubrication are similar to those for male penis erection [6].

The discovery of NO as a small signaling gasotransmitter led to the investigation of the biological roles of other endogenously derived gases, carbon monoxide (CO), hydrogen sulfide (H_2_S), sulfur dioxide (SO_2_), hydrogen (H_2_), and methane (CH_4_) in human body or in animals [21–33]. Among these gases, the effects of H_2_S [34–38] and CO [39–41] on sexual function and dysfunction have been extensively investigated. Mechanistically, sGC/cGMP pathway acts as one of the common target of these gasotransmitters during regulating penis erection [21]. In 2013, the protective effect of H_2_ on erectile dysfunction (ED) has also been confirmed [42]. Besides its role in ED, supplement of exogenous H_2_ has been shown to suppress testis injuries and improve sperm motility in male and also has the protective effects on female sexual organs. Therefore, the aim of this review is to summarize and discuss the effects and mechanisms of H_2_ in modulating sexual organs homeostasis, including the injury repair of sexual organs, fertility, and sexual function.

## 2. Basic Characteristics of H_2_

H_2_ has two different characteristics when compared with other five gases above: first, H_2_ is the lightest and diffusible gas molecule [43]; second, mammalian cells have no abilities to produce H_2_ due to lack of the functional hydrogenase genes [44]. The endogenous H_2_ in mammalian is mainly produced by hydrogenases-containing bacterial species located in gastrointestinal tracts (such as *Firmicutes* and *Bacteroidetes*), respiratory system (such as *Pseudomonas* and *Acinetobacter*), mouth and pharynx (such as *Eubacterium*), vagina (such as *Clostridium* species), and skin (such as *Corynebacterium*, *Acinetobacter*, and *Streptococcus*) [45]. H_2_ acts as a substrate for sulfate reducing bacteria, methanogenic bacteria, and acetic acid producing bacteria to utilize and support their energy metabolism [44, 46–48]. Therefore, the endogenous H_2_ levels in mammals are dependent on the balance between H_2_-producing fermentative bacteria and H_2_ consumers [44, 46–48]. In addition to being a source of energy for some bacteria, H_2_ has strong abilities to alleviate excessive oxidative stress basically by selectively reducing cytotoxic oxygen radicals, modulate immunity and inflammation, and suppress injuries-induced cell death [29, 30, 43, 49–52].

## 3. H_2_ Modulates Sexual Organs Homeostasis in Male

**Figure 1:**
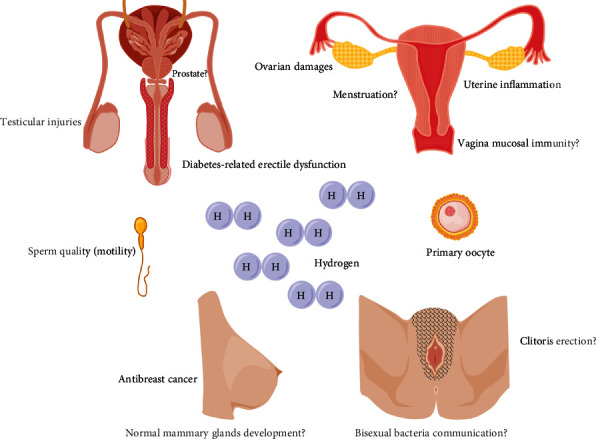
The target sex organs of H_2_ in animal models. In male animals, supplements of exogenous H_2_ may alleviate diabetes-related erectile dysfunction, various stresses-induced testicular injuries, and improve sperm quality (motility). In female animals, exogenous H_2_ has the protective effects on ovaries, uterus, and mammary glands, and improve the development of follicles. However, the functions of exogenous H_2_ in other sex organs, such as prostate in male, vagina and clitoris erection in female, the influence of bisexual bacteria communication in endogenous H_2_ levels, and the biological effects of exogenous H_2_ in sexual organs homeostasis, are not clear.

H_2_ is a novel bioactive gas molecule; it has essential roles in modulating male sexual organs homeostasis. Supplement of exogenous H_2_ has the protective effects on diabetes-related ED. Moreover, H_2_ attenuates numerous chemical, mechanical, and radiation damages-induced testicular injuries, modulates testosterone levels, and improves sperm quality (Figure 1, left).

### 3.1. H_2_ and Erectile Dysfunction

The penis is a vascular organ that is sensitive to changes in oxidative stress and systemic NO levels [9]. Vascular homeostasis maintenance is an active process, involved in the growth, migration, and death of vascular cells and activation of immune cells in vasculature, as well as the generation and degradation of extracellular matrix (ECM); all these coordinate with environmental cues to maintain the function of blood vessels [53]. H_2_ has strong abilities to suppress the excessive oxidative stress, thus maintaining vascular homeostasis and function, such as inhibiting abdominal aortic coarctation (AAC)- induced vascular hypertrophy and intimal hyperplasia in arterialized vein grafts in rats, decreasing blood pressure in monocrotaline-, N omega-Nitro-L-arginine methyl ester (L-NAME; NOS inhibitor)-, or chronic intermittent hypoxia-induced hypertension in rats [51, 54–57]. H_2_ also alleviates vascular dysfunction in spontaneous hypertensive rats (SHR) partially *via* enhancing NO bioavailability [58]. Fan et al. has investigated the effect of H_2_ on ED in a streptozotocin-induced diabetic rat model [42]. Compared with the diabetic rats, H_2_-rich saline gavage for 8 weeks improved ED, assessed by erectile frequency and intracavernous pressure measurement [42]. This protective effect of H_2_ on ED was related to reduce 8-hydroxy-2′-deoxyguanosine (8-OHdG) levels in serum and penis corpus cavernosum, attenuate malondialdehyde (MDA) levels and Bax expression in penis corpus cavernosum, elevate eNOS expression, NOS activity, NOx levels, and Bcl-2 expression in penis corpus cavernosum [42]. Therefore, the beneficial effects of H_2_ on penile erection are primarily mediated by suppressing oxidative stress and apoptosis and inducing NO production in corpus cavernosum.

### 3.2. H_2_ and Testicular Toxicity of Smoking

Approximately 37% of male adults worldwide use tobacco, mainly cigarettes [59]. Smoking looks like relaxation; however, tobacco smoke contains more than 4000 kinds of chemical compounds, such as nicotine, heavy metals (cadmium and lead), and benzo(a)pyrene [60]. Substantial harmful effects of cigarette smoke on fecundity and reproduction have become apparent but are not generally appreciated [61]. Moreover, tobacco smoking is scientifically recognized as a risk factor of erectile impotence [60]. Oxidative stress and the resulting genetic and epigenetic changes that result from smoking may correlate directly with reduced sperm function and reduced fertility [60, 62]. Using cigarette smoke rat model, Chen et al. revealed that H_2_ subcutaneous injection increased the sperm count, increased serum testosterone levels, decreased the upregulation of sperm deformation rate and testicular MDA levels, and increased testicular and serum SOD activities [63]. Similarly, H_2_-rich saline decreased testicular and serum MDA levels, testicular H_2_O_2_, nitrotyrosine, and protein carbonyl levels; decreased testicular Caspase-3 activity; significantly increased both testicular and serum testosterone levels; and increased in sperm number and motility in mice subjected to chronic nicotine treatment [64]. However, whether H_2_ has the beneficial effects on cigarette smoke-related ED is not clear.

### 3.3. H_2_ and Spinal Cord Hemisection-Induced Testicular Injury

Most men with spinal cord injury (SCI) are infertile [65]. ED, ejaculatory dysfunction, and semen abnormalities contribute to the problem [65]. SCI is a highly inflammatory process that affects multiple organs that we do not fully understand, including the testis [66, 67]. Moreover, reactive oxygen species (ROS) are higher in SCI men, and there is an inverse correlation between the level of ROS and sperm motility [67, 68]. Ge et al. investigated the effects of H_2_-rich saline on the testicular biological function by establishing a hemi-sectioned spinal cord injury (hSCI) rat model (laminectomy at the T10-T12 level); they found that H_2_-rich saline upregulated the reduced ratio of testis weight/body weight, attenuated testicular morphological injury, improved the ultrastructural damage of cells in testis, increased the downregulated mean seminiferous tubular diameter and seminiferous epithelial thickness, reduced apoptosis of spermatogenic cells, increased testicular mitofusin-2 (an outer mitochondrial membrane GTPase involves in mitochondrial fusion and endoplasmic reticulum-mitochondria tethering [69]), and decreased testicular heme oxygenase-1 (HO-1, an enzyme catalyzes oxidation of heme to biologically active molecules: iron, a gene regulator; biliverdin, an antioxidant; and CO, a heme ligand [70]) and high-mobility group box 1 (HMGB-1, an endogenous danger signal and inflammatory mediator) levels in rats subjected to hSCI [71]. However, the influences of H_2_ on the impaired erectile function after SCI still need further investigation.

### 3.4. H_2_ and Testicular Torsion

Testicular torsion is a true urological emergency most commonly seen in adolescence, which has been estimated to affect 1 in every 4 000 males younger [72–74]. Testicular ischemia/reperfusion (I/R) caused by the twisting and release of the spermatic cord can result in biochemical and morphological changes; these have long-term effects on fertility and result in testicular atrophy, even if the testis can be salvaged [72, 73]. The mechanism of testicular injury through reperfusion involves neutrophil recruitment, generation of ROS and reactive nitrogen species (RNS), proinflammatory cytokines and adhesion molecules, lipid peroxidation, apoptosis, anoxia, and alteration to microvascular blood flow [72, 73]. Inhalation of 2% H_2_ has the therapeutic effects on testicular I/R injury in rats as indicated by attenuating abnormal morphology and the impaired spermatogenesis, decreasing germ cell apoptosis and testicular MDA levels [75]. Moreover, H_2_-rich saline injection normalized lipid peroxidation levels and preserved activity of SOD, thus reducing testicular I/R injury score and apoptosis index [72, 76]. These studies confirmed the antioxidant, anti-inflammatory, and antiapoptotic effects of H_2_ on testicular I/R injury.

### 3.5. H_2_ and Testicular Damage Caused by Radiation

Nuclear technologies utilization in power production, medicine, and industrial production drive the progress of modern civilization and make a better life; however, people exposed to ionizing radiation have the potential health threats [77, 78]. Ionizing radiation induced oxidative stress, epigenetic changes and genomic instability, and disturbed mitochondria, thus inducing the detrimental effects on organisms [79–82]. As one of the most radiosensitive organs, testicular function can be significantly impaired by ionizing radiation exposure [83]. H_2_-rich saline attenuated *γ*-radiation-induced testis damage through reducing testicular hydroxyl radicals (·OH), MDA, protein carbonyl, and 8-OHdG levels, while restoring serum testosterone levels, and SOD and GSH levels in testis, attenuating apoptosis of spermatogenic cells, preserving stem spermatogonia survival, and the daily sperm production and sperm quality [78, 84]. This provides the evidence that H_2_ has potential clinical applications in preventing testicular damage caused by radiation.

### 3.6. H_2_ and Sperm Motility

The average life expectancy has been increasing, and many aging men have the need to maintain normal sexual function, as well as their fecundity [85]. Aging-related oxidative stress plays a crucial role in the progression of age-related male infertility [85]. As we have discussed above, H_2_ improved sperm quality after testicular damage *in vivo*. The protective effect of H_2_ on sperm motility was further confirmed by human sperm *in vitro*; it improved sperm motility of experimentally damaged sperm suspensions from patients left at room temperature for >5 days or frozen immediately after ejaculation, and increased mitochondrial membrane potential; however, H_2_ treatment did not affect sperm swimming speed [86]. Therefore, H_2_ is a new promising tool for male infertility treatments [86].

Varicocele-induced male infertility potentially involves oxidative stress [87]. Recently, Inagaki developed a silicon-based agent that produces H_2_ by the reaction with water [87–89]. By using this agent, they have investigated the therapeutic effects of H_2_ on a varicocele rat model. They found that oral intake of the silicon-based agent improves epididymal sperm motility and *in vitro* fertilization rates *via* H_2_ production and subsequent reduction of oxidative stress [87]. The protective effect of H_2_ on sperm was also confirmed in normal young and aged male mice. Ku et al. found that H_2_-rich water or Korean Red Ginseng treatment by gavage stimulated spermatogenesis followed by increasing sperm motility in 3-month-old male mice and increasing sperm count and sperm motility in 12-month-old male mice [85]. These effects were strengthened synergistically by the H_2_-rich Korean Red Ginseng water mixture [85]. These functional water has the abilities to modulate the expressions of antioxidation (PPx3, PPx4, GSTm5, and GPx4), spermatogenesis (inhibin-*α*, neptin-2 and cyclic AMP responsive element modulator (CREB)), antiaging (sirtuin 1 (SIRT1) and SIRT2), and angiogenesis (visfatin and vascular endothelial growth factor (VEGF)) related genes in the testes and decrease serum ROS level [85]. The serum testosterone levels were increased in both young and old mice after drinking 4 weeks of H_2_-rich water; the serum follicle-stimulating hormone (FSH) levels were increased only in old mice receiving H_2_-rich water [85]. However, the levels of FSH as well as of luteinizing hormone (LH) were not significantly influenced in rats after 4 days of receiving a single dose of *γ*-irradiation or treatment with H_2_-rich saline [84]. The difference of these two studies might be related to the time and forms of H_2_ used, as 4 days are too short to influence hormones levels, and might also be related to the animal model used.

## 4. H_2_ Modulates Sexual Organs Homeostasis in Female

In female animal models, supplements of the exogenous H_2_ also display essential roles in modulating sexual organs homeostasis. H_2_ may alleviate I/R- and drug-induced ovarian injuries; improve postmenopausal osteoporosis, premature ovarian failure (POF), and follicles development; reduce uterine inflammation; ameliorate several characteristics of preeclampsia; and has the antibreast cancer effect (Figure 1, right).

### 4.1. H_2_ and Adnexal Torsion

Adnexal torsion is a gynecologic disorder caused by the partial or complete twist of the ovary and/or the fallopian tube on the axis created between the infundibulopelvic ligament and the utero-ovarian ligament [90, 91]. It usually presents as a sudden, continuous, nonspecific pain in the lower abdomen, and the annual prevalence is approximately 2% to 6% [91, 92]. As that ischemia is the direct consequence of the twists in the adnexa, therefore, laparoscopic detorsion should be performed in order to preserve the integrity of the ovaries and fertility; nevertheless, detorsion creates I/R injury, which causes ovarian damage through the induction of oxidative stress, inflammation, and apoptosis [93–95]. H_2_-rich saline attenuated follicular injury, edema, hemorrhage, loss of cohesion, and the upregulation of apoptotic index in ovarian I/R rats model *via* modulating MDA and glutathione-S-transferase (GST) levels [96]. Therefore, H_2_ is a novel bioactive gas molecule in attenuating I/R-induced ovary injury.

### 4.2. H_2_ and Osteoporosis after Menopause

Menopause is defined as the permanent cessation of ovulation and menstruation due to ovarian failure [97]. The menopause, as measured by the last menstrual period, occurs at an average age of 50.7 years [98]. The estrogen (E_2_) level after menopause is inadequate to maintain E_2_-dependent tissues, leads to the gradually atrophy of breasts, vulvar and vaginal, thinning and dryness of the vaginal epithelium, and osteoporosis [99–101].

Oxidative stress plays an essential role in the progression of osteoporosis [102, 103]. The animal study indicated that H_2_ has the beneficial effect on osteoporosis after menopause [104, 105]. Daily treatment with 60% H_2_ protected against postmenopausal osteoporosis in an ovariectomized (OVX) mice model by reducing serum levels of proinflammatory cytokine IL-1*β*, IL-6, and TNF-*α* [105]. Moreover, H_2_-rich water consumption prevented osteopenia in OVX rats, while had no significant effect on plasma E_2_ levels [104]. The plasma levels of NOx, which is the stable end products of NO, and femur eNOS mRNA levels were increased by H_2_ in ovariectomized rats, indicated that H_2_ can induce NO production [104]. However, if H_2_ can improve atrophy of breasts, vulvar, and vaginal, and thinning and dryness of the vaginal epithelium after menopause are not clear.

### 4.3. H_2_ and Premature Ovarian Failure

Menopause before the age of 40 years is defined as premature menopause, also known as POF, which occurs in 1% of women [106–111]. POF is characterized by amenorrhoea, hypo-oestrogenism, and elevated gonadotrophin levels due to cessation of ovarian function [106–110]. Genetic aberrations, autoimmune ovarian damage, latrogenic following surgical, radiotherapeutic or chemotherapeutic interventions as in malignancies, and environmental factors like viral infections and biohazardous environmental chemicals, etc., are responsible for the pathogenesis of POF [106, 112–116]. H_2_ has been shown to protect against POF and the drug-induced ovarian injury [117, 118]. Drinking H_2_-rich water increased serum anti-Müllerian hormone (acting as a marker in POF and a good predictor of the time of menopause [119]) levels and ovarian Bcl-2 expression, decreased granulosa cell apoptotic index, and Bax/Bcl-2 ratio in a POF mice model induced by immunized with zona pellucida glycoprotein 3; these indicated that H_2_ exerted protective effect on ovarian reserve function in mice with immune POF [117]. In a cisplatin-induced ovarian injury rat model, H_2_-rich saline attenuated the serum follicle-stimulating hormone (FSH) release, elevated the serum level of E_2_, improved the development of follicles, and reduced the damage to the ovarian cortex [118]. This protective effect of H_2_-rich saline on ovarian injury is involved in increasing the activities of SOD and catalase and reducing the level of MDA in the serum and ovarian tissue and increasing ovarian nuclear factor erythroid 2-related factor 2 (Nrf2) expression [118]. Future studies should focus on whether H_2_ can modulate the immune dysfunction in POF animal models.

### 4.4. H_2_ and Uterine Inflammation

Intrauterine inflammation causes preterm birth and is associated with complications in preterm neonates [120, 121]. Nakano et al. found that drinking H_2_-rich water may significantly prevent uterine inflammation *via* reducing the expression of proinflammatory cytokines (*Tnf* and *Il6*), contractile-associated proteins (*Cox2* (*Ptgs2*)*, Cx43* (*Gja1*), and *Oxtr*), and *Et1* (*Edn1*) in the uterus, increases the levels of progesterone in the maternal serum, potentially extending the duration of pregnancy in a murine model of lipopolysaccharide (LPS)-induced preterm birth [121]. Their group also found that drinking H_2_-rich water ameliorates several characteristics of preeclampsia in the reduced uterine perfusion pressure (RUPP) rat model, such as decreasing mean arterial pressure, increasing fetal and placental weight, and attenuating angiogenic imbalance and oxidative stress [122]. Additionally, it is interesting to investigate the influence of H_2_ on menstruation.

### 4.5. H_2_ and Breast Cancer

Drinking the electrochemically reduced water, which is rich in H_2_, may delay mammary tumors growth in mice and inhibit the survival and induce apoptosis of human (MCF-7 and MDA-MB-453) and mouse (TUBO) breast cancer cell lines *in vitro* [123]. Another study indicated that combination of ammonia borane-mediated H_2_ therapy and polydopamine-mediated photothermal therapy may maximize the therapeutic effects on breast cancer in mice and overcome undesirable proinflammatory responses [124]. However, the effects of H_2_ on the normal growth and development of mammary glands are not clear.

## 5. Perspective

### 5.1. Gases Interaction in Modulating Sexual Organs Homeostasis

NO, CO, and H_2_S are key gas molecules contributing to penis erection [5, 34, 35, 39, 41, 125]. H_2_S and H_2_ can induce NO production in corpus cavernosum [42, 126]. Moreover, the exogenous CO can induce relaxation of phenylephrine precontracted corpus cavernosum smooth muscle in response to electrical field stimulation [39]. This relaxation may be mediated by CO-dependent activation of sGC and subsequent elevation of cGMP or CO-dependent induction of NO release [39, 127, 128]. H_2_ is primarily produced by hydrogenase-expressing fermentative bacteria in human body; H_2_S and CH_4_ are the by-products of H_2_ metabolism derived from sulfate-reducing bacteria and methanogenic bacteria, respectively [44]. Therefore, the role of intestinal flora-derived H_2_S should also be taken into consideration when discussing sex organs homeostasis. It is well known that mammalian cells can also produce H_2_S and CH_4_ by its own enzyme system [26, 31, 129]. It is not clear whether exogenous or endogenous H_2_ can modulate CO, H_2_S, or CH_4_ production in corpus cavernosum. CH_4_ has anti-inflammatory effects, and numerous studies have shown the involvement of inflammatory processes in male and female sexual dysfunction [6, 130]. The function of CH_4_ in sexual dysfunction is not clear, if exist, whether the key molecular mechanism of CH_4_ is mediated by the classical NO, H_2_S, or CO signal, or by other unknown targets? Is this effect influenced by the endogenous or exogenous H_2_? These endogenous gas molecules always exist as the gas mixture; they may arrive at the sexual organs by free diffusion or by blood circulation, thus influencing sexual organs homeostasis, respectively, together by another one or more. Therefore, there exist the gases interaction networks among NO, CO, H_2_S, H_2_, and CH_4_, and these networks might play the essential roles in modulating sexual organs homeostasis.

### 5.2. Interaction between Sexual Activities and Endogenous H_2_

The sexual activities between male and female always involve emotional communication, kissing and saliva exchange, petting, touching genitals, oral sex (fellatio (oral-penile contact), cunnilingus (oral-vaginal contact), and analingus (oral-anal contact)), anal sex, coitus, etc. [131–137]. Kissing and saliva exchange can transmit oral microbiota [138], and oral sex can also transmit oral, respiratory, and genital infections from one site in body to the other [133, 135]. Human lower reproductive tract (vagina, cervix, and penile urethra) is the direct place for sexual intercourse; the mucosal epithelia in these organs are exposed to sexually transmitted microbes [139, 140]. As we have discussed above, the endogenous H_2_ is mainly produced in the gastrointestinal tracts, mouth and pharynx, vagina, respiratory system, and skin by H_2_-producing fermentative bacteria [45]. Therefore, sexual activities will promote the bisexual bacteria communication, including the bacteria in the organs involved in sexual activities, and the endogenous H_2_ levels might be easily affected by sexual activities. However, the biological functions of this transfer in sex activities are not clear.

### 5.3. The Endogenous H_2_ in Sexual Organs Homeostasis

The endogenous H_2_ is the product of the metabolism of anaerobic bacteria (H_2_-producing fermentative bacteria) by degrading the unabsorbed hydrocarbons in the intestinal tract of mammals [30, 44]. Therefore, diet can influence the production of endogenous H_2_. For example, H_2_ was produced in greater amounts by breast-fed infants than by infants feeding with a soy-based or a milk-based formula, presumably the consequence of incomplete absorption of breast milk oligosaccharides [141]. Another clinical trial in Japan indicated that curcumin can activate carbohydrate colonic fermentation in human, thereby increasing the concentration of breath H_2_ [142]. In animals, lactulose, L-arabinose, and diet with 20% high amylose cornstarch can enhance the production of endogenous H_2_; the produced endogenous H_2_ is an essential regulator of liver homeostasis, such as improving hepatic I/R injury, liver regeneration, and hepatic steatosis as well as glucose and lipids homeostasis [44, 143–145]. The exogenous H_2_ has protective effects on testes damages and alleviates ED in a diabetic rat model; in female animal disease models, exogenous H_2_ has the antibreast cancer effects and protects ovaries and uterus against injuries (Figure 1). However, little is known about the physiological function of endogenous H_2_ in sexual organs homeostasis, such as penis and clitoral erection, and the influence of endogenous H_2_ in reproductive tract, for example, its roles in vagina, and mouth and pharynx mucosal immunity are also charming (Figure 1).

## Data Availability

The data used to support the findings of this study are included within the article.
